# A BERT-based pretraining model for extracting molecular structural information from a SMILES sequence

**DOI:** 10.1186/s13321-024-00848-7

**Published:** 2024-06-19

**Authors:** Xiaofan Zheng, Yoichi Tomiura

**Affiliations:** Graduate School of Information Science and Electrical Engineering, Department of Informatics, Kyushu University, Fukuoka, Japan

**Keywords:** SMILES, ADMET molecular properties prediction, Odor descriptors, Transformer model, BERT, Pretraining

## Abstract

**Abstract:**

Among the various molecular properties and their combinations, it is a costly process to obtain the desired molecular properties through theory or experiment. Using machine learning to analyze molecular structure features and to predict molecular properties is a potentially efficient alternative for accelerating the prediction of molecular properties. In this study, we analyze molecular properties through the molecular structure from the perspective of machine learning. We use SMILES sequences as inputs to an artificial neural network in extracting molecular structural features and predicting molecular properties. A SMILES sequence comprises symbols representing molecular structures. To address the problem that a SMILES sequence is different from actual molecular structural data, we propose a pretraining model for a SMILES sequence based on the BERT model, which is widely used in natural language processing, such that the model learns to extract the molecular structural information contained in the SMILES sequence. In an experiment, we first pretrain the proposed model with 100,000 SMILES sequences and then use the pretrained model to predict molecular properties on 22 data sets and the odor characteristics of molecules (98 types of odor descriptor). The experimental results show that our proposed pretraining model effectively improves the performance of molecular property prediction

**Scientific contribution:**

The 2-encoder pretraining is proposed by focusing on the lower dependency of symbols to the contextual environment in a SMILES than one in a natural language sentence and the corresponding of one compound to multiple SMILES sequences. The model pretrained with 2-encoder shows higher robustness in tasks of molecular properties prediction compared to BERT which is adept at natural language.

**Supplementary Information:**

The online version contains supplementary material available at 10.1186/s13321-024-00848-7.

## Introduction

Molecules as microscopic units constitute macroscopic matter, and their properties directly affect the application of substances in our daily lives. Depending on the direction of application, we require different chemical and physical properties of molecules, including simple properties such as hydrophilicity and complex properties such as protein binding. The factors that affect these properties can be traced back to deeper physical principles, but the computational cost is huge for multi-particle systems. It takes a long time to obtain complex molecular properties by adopting either experimental or computational chemistry methods. Obtaining molecular properties through machine learning is thus being considered.

Machine learning methods have been widely applied in chemistry, biology, and material informatics. Machine learning approaches have been proposed for the prediction of chemical properties [1, 2], the synthesis of compounds [3, 4], and the prediction of chemical reaction products [5]. Although molecular properties are diverse, the factors that determine their properties often depend on some common key factors such as the hydrophilicity of the molecule, whether it contains certain functional groups, etc. These common key factors of molecules can be quickly calculated, consequently, models such as random forest (RF) with inputs of molecular fingerprints and molecular descriptors often perform well in predicting molecular properties (even when the data size is small) as shown in [6]. In addition to using feature-based methods to infer unknown molecular properties, people also attempt to directly summarize features from molecular structures with artificial neural networks to infer molecular properties. We can roughly divided these feature-free methods of extracting features of molecular structures and predicting molecular properties using artificial neural networks into three categories according to the type of inputs to the model. The first category uses a SMILES (simplified molecular-input line-entry system) sequence as the input to the model. A SMILES sequence comprises symbols representing the molecular structure. The atoms that appear in a molecule are expressed by the symbol of their atom type, the substructure of a branch chain is represented in brackets ’()’, and the ring structure is represented by adding the same number after the start atom and end atom of the ring. A SMILES sequence can also represent stereo structures using ’$\backslash$’ and ’/’ for the isomers due to double bonds and using ’@’ and ’@@’ for optical isomers. As SMILES sequences can be regarded as having the same data structure as a sentence, most works deal with SMILES sequences using a model developed for natural language. [7] proposed a model based on long short-term memory to predict molecular properties and interpreted the results with an attention mechanism. [8–11] applied the BERT model [12] to pretrain and predict molecular properties. [13] used fingerprints converted according to SMILES as inputs and pretrained the model with BERT. In addition, models can be pretrained with SMILES sequences in a language translation fashion by leveraging the fact that different SMILES sequences can represent the same molecular structure. [5, 14] pretrained a model by translating a SMILES sequence to a different SMILES sequence (where the SMILES sequences represent the same molecule) with a transformer model. The second category uses graph data as input to the model. Atoms in a molecule are represented as nodes, and chemical bonds are represented as edges. The models using graph data as input are mostly graph neural network models. Nodes in a graph pass information through edges, and the graph neural network [1, 15–17] can be used to learn the representation of individual nodes as well as the representation of the whole graph. The third category uses the three-dimensional (3D) geometric structure of molecules as the input of the model [18–20]. The inputs of the model include atom types, distances between atoms, and angular relationships. This type of model is usually used in a manner similar to the methods used in computational chemistry and is often used to predict more fundamental molecular properties, such as the potential energy.

In evaluating the performance of the proposed pretraining model, we predict 22 molecular properties in 22 therapeutic datasets by finetuning the pretrained model in the experimental part of this study. In addition to the 22 molecular properties, we are particularly interested in the odor characteristics of molecules. Unlike the case for other senses, such as vision and hearing, the mechanism of olfaction has not been elucidated. We expect to obtain the representation of the odor properties of molecules that predict odor descriptors (ODs) manually labeled to them using artificial neural networks. Studies using a molecular structure to predict ODs [21–23] predicted few (no more than 20) ODs using feature-based methods, such as the support vector machine and random forest methods. [24] combined two data sets to predict 138 ODs with a graph neural network and achieved an F1 value of 0.38, and they clustered ODs with the previous layer of the output layer. Our previous study [25] used one data set to predict 98 ODs with a transformer model and achieved an F1 value of 0.36. However, the model in [25] was designed to obtain the representation for each OD individually in interpreting the substructures that affect it, which does not provide a representation of overall odor properties.

This paper focuses on the pretraining embeddings of molecules that predict various molecular properties. We believe that a simple application of the masked language model (MLM) BERT, which is a pretraining model used in natural language processing, is insufficient. The contributions of this research are

- the proposal of a model for pretraining embeddings of SMILES representation,
- the prediction of molecular properties in 22 data sets to evaluate the effectiveness of the proposed pretraining model, and
- obtaining the odor representation of molecules by predicting 98 kinds of OD.

## Method and experiment

### Method

In this study, we used SMILES sequences as representations of molecular structures for model input. Compared with inputs in the form of a graph and 3D geometric structure, a SMILES sequence does not express the relationship between atoms as straightforwardly as adjacency and distance matrices. It is thus necessary for the model to learn the structural information implied in the SMILES sequence. Fortunately, this can be done through unsupervised learning without manually labeled data. As we mentioned in the Introduction, a SMILES sequence can represent stereoisomers explicitly with the symbols ’/’, ’$\backslash$’, ’@’, and ’@@’. Specifically, the trans and cis isomers caused by double bonds can be identified by the directions of symbols ’/’ and ’$\backslash$’, and the chirality of optical isomers can be identified by ’@’ and ’@@’. On the contrary, identifying stereoisomers is more complicate with adjacency and distance matrices. Adjacency matrices cannot identify stereoisomers at all. Although the difference in stereo structure is reflected in the distance between atoms, it requires positional relationships between at least three atoms, and the interference between atoms decays rapidly with increasing distance. Therefore, it may be more difficult to make the model learn the difference between stereoisomers by distance. At this point, using a SMILES sequence as the input of the model can be regarded as better than representing stereoisomers using adjacency matrices and distance matrices.

SMILES sequences can be treated as sentences in natural language. In recent years, the transformer model has been used in natural language processing with great success. The original transformer model [26] was used for machine translation. The final tasks for natural language, such as semantic analysis and dialogue generation, are varied. However, regardless of the specific task, the grammar and meaning of the words remain the same, and learning this invariant knowledge in a language through pretraining is thus of great help to specific tasks such as prediction and generation. The BERT model [12] is one of the pretraining models based on the transformer model. In this paper, we propose a model for pretraining embeddings of SMILES representation that improves on the BERT model, and we then fine-tune the pretrained model to predict molecular properties.

**Fig. 1:**
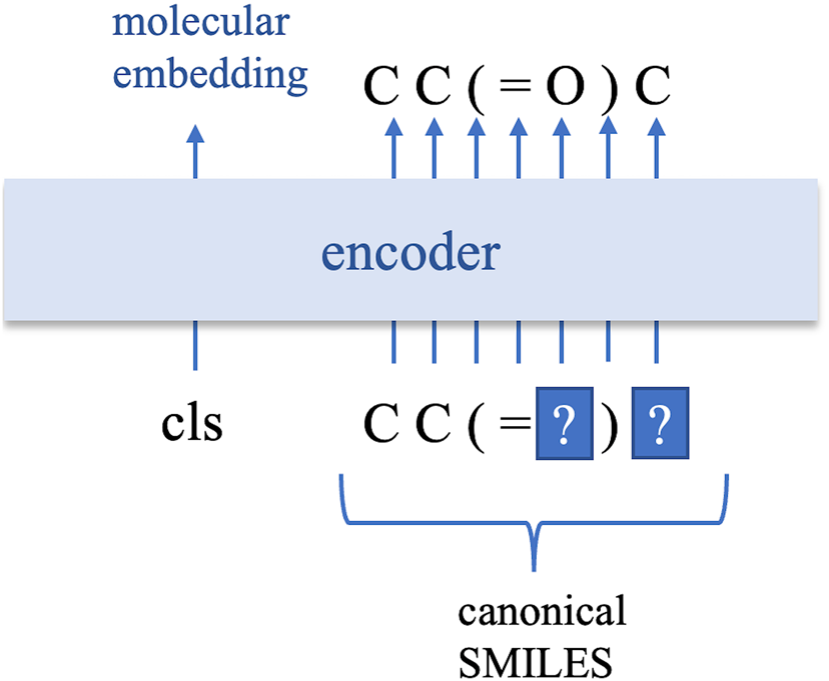
BERT MLM pretraining model

In the paper of BERT [12], the model was pretrained on two tasks, namely the next sentence prediction (NSP) and MLM tasks. The NSP task is to predict whether two sentences are consecutive. As there is no consecutive relationship between two SMILES sequences, we did not pretrain our model on the NSP task. The MLM task is to recover the words that are masked randomly in the input sentence. As shown in Fig. 1, [8] applied the BERT MLM to SMILES sequences directly. It is possible for the BERT MLM to recover partial symbols of masked SMILES by learning the grammar implied in the SMILES sequence, such as the equal numbers of ’(’ and ’)’ and the possible number of branches of an atom type. However, some of the atoms or substructures in a molecule are not definitely related to the remaining part of the molecule. For example, an atom bonded to a carbon atom can be any of multiple types of atom instead of being limited to a certain type of atom, and the BERT MLM tends to recover the symbol corresponding to the atom most frequently occurring, such as ’C’. It is thus impossible for the BERT MLM to recover masked SMILES sequences exactly. Moreover, it is difficult to recover masked SMILES sequences when the masking rate is set higher than 10% ~20%, which limits the benefits of pretraining.

**Fig. 2:**
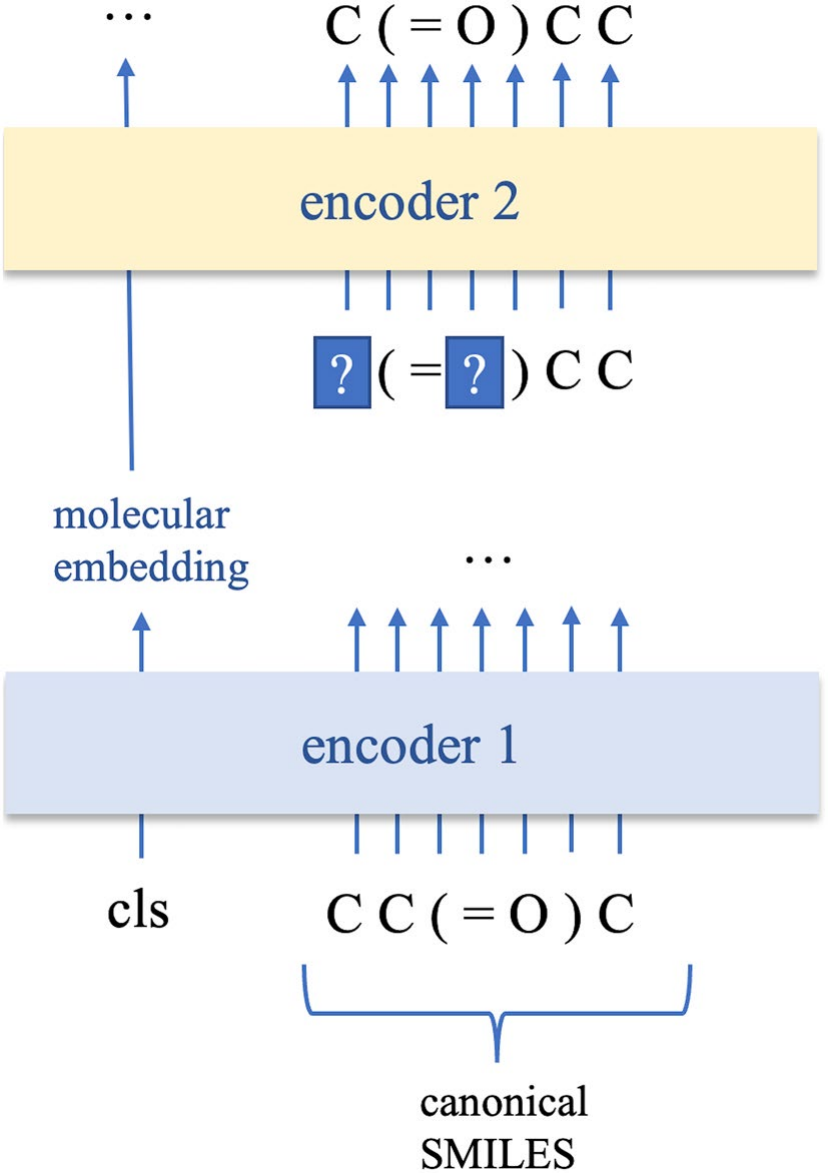
2-encoder pretraining model

To solve the above problem, in this paper, we propose a pretraining model as shown in Fig. 2. The model comprises two encoders. We refer to the proposed model as the 2-encoder model in the remainder of the paper. The inputs of the first encoder are the canonical SMILES sequence and a special character called ’cls’. Through the first encoder, the output corresponding to ’cls’ is regarded as the molecular embedding, and it is one of the inputs to the second encoder. In addition to molecular embedding, a SMILES sequence (which represents the same molecule corresponding to the SMILES input to the first encoder) masked randomly is input to the second encoder, and the output of the second encoder is the recovered SMILES sequence corresponding to the masked SMILES sequence. For the 2-encoder model, as the input SMILES sequence to the first encoder is not masked, we assume that the molecular embedding contains the exact information of the entire SMILES sequence and has the ability to recover the masked SMILES sequence input to the second encoder accurately. A molecule can be represented by different SMILES sequences; e.g., CC(=O)C and C(=O)CC represent the same molecular structure. To avoid the molecular embedding obtained from the first encoder memorizing only the input SMILES sequence rather than the molecular structural information, approximately 80% of the SMILES sequence input to the second encoder is different from the SMILES sequence input to the first encoder. By avoiding the problem in the BERT MLM that the masked symbol cannot be fully recovered, the molecular embedding obtained using the 2-encoder pretraining model is expected to contain the whole molecular structure implied in the SMILES sequence.

**Fig. 3:**
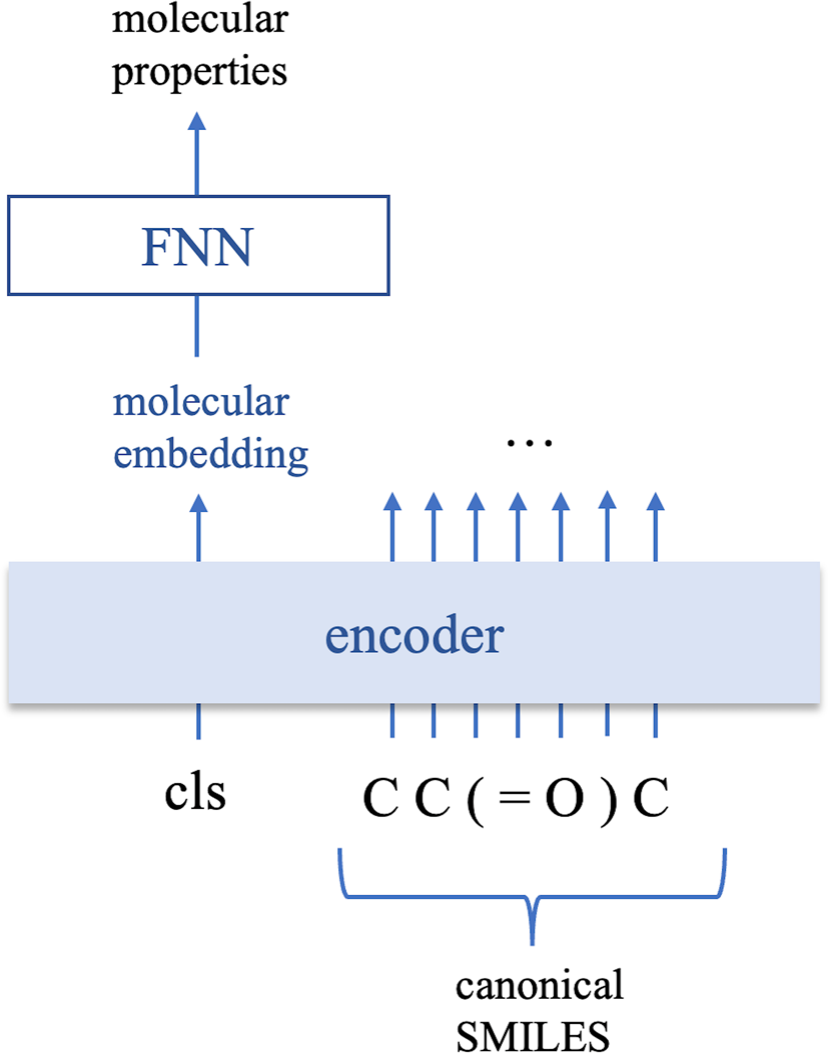
Model used to predict molecular properties with the input of the SMILES sequence

In the stage of predicting molecular properties, only the first encoder in the 2-encoder model is kept; i.e., we add a fully connected neural network after the molecular embedding to predict molecular properties as shown in Fig. 3.

Our proposed 2-encoder model differs from the BERT model [8–11] in two main points.

- In the second encoder, masked symbols can be uniquely determined since molecular embeddings are one of inputs which already include information of canonical SMILES representing the same molecule. And the mask rate of 2-encoder model is 50% compared with 15% of the BERT model in pretraining model with MLM task.
- A meaningful molecular embedding is achieved in the pretraining stage by using 2-encoder model, and this may thus reduce the burden of summarizing molecular embeddings during the finetuning stage.

### Experiment

In evaluating the performance of the 2-encoder pretraining model, we first pretrained the model by recovering the masked symbols in the SMILES sequence and then used the pretrained model to predict molecular properties.

In the pretraining stage, we trained the BERT MLM shown in Fig. 1 and our proposed 2-encoder model shown in Fig. 2. (More specifically, the model structure of BERT MLM we used should be the same as that of [8, 10, 11], but the tokens for inputs of encoder are different from [10, 11]. As shown in Fig. 1, the tokens used for our BERT MLM and [8] are each symbol appearing in a SMILES, whereas [10, 11] generated tokens with Byte-Pair Encoder. ) Although the model structures of the BERT MLM and 2-encoder are different in the pretraining stage, they are the same in the fine-tuning stage because the 2-encoder model only keeps the first encoder to predict molecular properties. In addition, to evaluate the effectiveness of pretraining, we trained the model shown in Fig. 3 directly to predict the molecular properties. We refer to this model as the non-pretrained model in this paper. We used RDKit to compute the inputs of models including converting the canonical SMILES sequence to different SMILES sequences expressing the same molecular for the 2-encoder model.

**Table 1:** Hyperparameter settings of pretraining

	BERT MLM	2-encoder
Number of encoder layers	8, 10	8, 10
Number of heads	8, 16	8, 16
Dimension of molecular embedding	128, 256	128, 256
Mask rate	0.1	0.5
Learning rate	0.0003	0.0003
dropout rate	0.1	0.1

The data set used in the pretraining stage was a SMILES data set of 100,000 molecules collected from ChEMBL [27] (with the SMILES sequence length not exceeding 100). Table 1 gives the hyperparameter settings that we used in the pretraining stage.

**Table 2:** Number of SMILES with a length of exceeding 100

	Sample size	Number of exceeding 100
ames	7278	28
bbb_martins	2030	98
bioavailability_ma	640	35
caco2_wang	910	89
clearance_hepatocyte_az	1213	17
clearance_microsome_az	1102	3
cyp2c9_substrate_carbonmangels	669	36
cyp2c9_veith	12092	259
cyp2d6_substrate_carbonmangels	667	36
cyp2d6_veith	13130	256
cyp3a4_substrate_carbonmangels	670	38
cyp3a4_veith	12328	232
dili	475	15
half_life_obach	667	76
herg	655	17
hia_hou	578	26
ld50_zhu	7385	23
lipophilicity_astrazeneca	4200	11
pgp_broccatelli	1218	39
ppbr_az	2790	26
solubility_aqsoldb	9982	225
vdss_lombardo	1130	94

In this study, we evaluated the performance of our model on data sets provided by Therapeutics Data Commons (TDC) [28]. TDC provides a variety of data sets for machine learning, including molecular property prediction, pairwise molecular reaction prediction, and compound generation. In addition to data sets, TDC also provides leaderboards, where people can upload experimental results of their own models to facilitate comparison of different models. We predicted molecular properties of the benchmark group of the ADMET properties (absorption, distribution, metabolism, excretion and toxicity properties) in the leaderboards.The ADMET group contains 22 datasets (6 datasets of absorption properties namely caco2_wang, bioavailability_ma, lipophilicity_astrazeneca, solubility_aqsoldb, hia_hou and pgp_broccatelli; 3 datasets of distribution properties namely bbb_martins, ppbr_az and vdss_lombardo; 6 datasets of metabolism namely cyp2c9_veith, cyp2d6_veith, cyp3a4_veith, cyp2c9_substrate_carbonmangels, cyp2d6_substrate_carbonmangels and cyp3a4_substrate_carbonmangels; 3 datasets of excretion namely half_life_obach, clearance_hepatocyte_az and clearance_microsome_az; 4 datasets of toxicity namely ld50_zhu, herg, ames and dili). We loaded datasets and evaluated prediction results with functions provided by TDC so that the obtained results can be directly compared with the results recorded on the leaderboards. Since our pretrained model requires that the length of SMILES does not exceed 100 due to the size of GPU we used, we simply truncated the first 100 symbols and used truncated SMILES as the inputs of the model. The details of number of SMILES that length exceed 100 for 22 datasets can be found in Table 2. Symbols that do not appear in the data set of pretraining are replaced with the ID of the mask.

In addition, we predicted the odor characteristics of molecules; i.e., ODs. It is difficult to compare different models on predicting ODs since there is no free and publicly available dataset for hundreds of odor characteristics of molecules. Nevertheless, since molecular odor properties may depend on a variety of complex factors such as local substructure, overall molecular shape, etc., we think it is worth reporting the performance of the proposed model in predicting ODs in this paper. We used the same data set as used in our previous study [25], which was provided by TheGoodScentsCompany [29]. This data set had 4365 samples, and each molecule was manually labeled with ODs, such as the terms fruity and sweet. For most ODs, only a small number of samples were labeled positive in the data set. There were nine ODs with more than 400 positive samples, namely fruity, sweet, green, floral, woody, herbaceous, fresh, fatty, and spicy. We predicted 98 ODs with more than 50 positive samples in the experiment.

In the experiment on the prediction of molecular properties, we adopted a cross-validation approach and took the average results for comparison of the different models. For the pretrained BERT MLM and 2-encoder models, we tried three ways to fine-tune them for predicting specific molecular properties: training only the last two encoder layers and the last fully connected layer, training only the last encoder layer and the last fully connected layer, and training only the last fully connected layer. The learning rate was set at 0.001, 0.0007, and 0.0001 respectively.

## Results and discussion

### Results of pretraining

For both the BERT MLM and the 2-encoder model, the best accuracy of symbol recovery was achieved when setting hyperparameters of the dimension of molecular embedding to 256, and the accuracy is hardly affected by the hyperparameters of the number of encoder layers and the number of heads. The accuracy achieved by the 2-encoder model was 0.98 and that achieved by the BERT MLM was 0.92. The results show that even though the mask rate set for the 2-encoder model was higher than that set for the BERT MLM, the 2-encoder model still achieved a better accuracy of recovery.

**Fig. 4:**
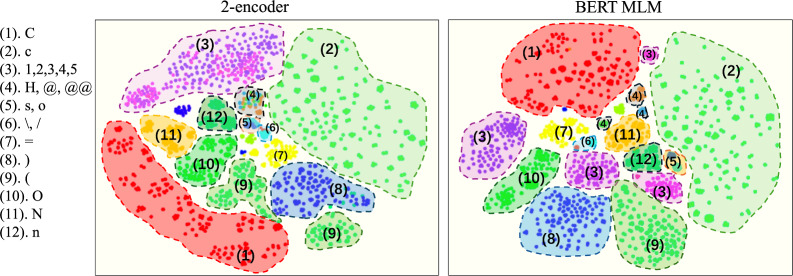
Comparison of symbol embedding

**Fig. 5:**
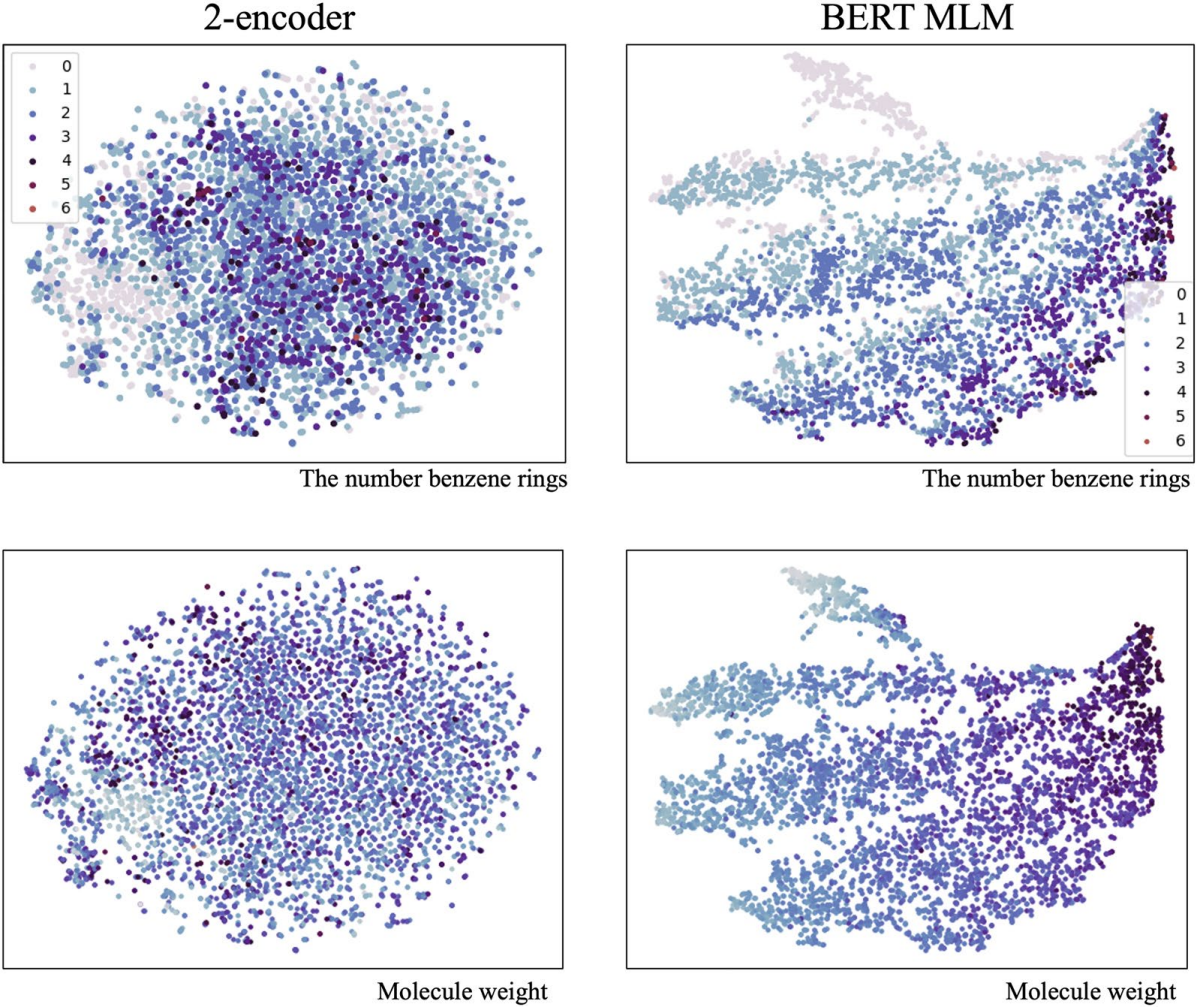
Comparison of molecular embedding of the 2-encoder and BERT MLM

To compare embedding obtained by these two kinds of pretrained model, 5000 canonical SMILES are chosen randomly and their corresponding outputs of the first encoder in the 2-encoder model and the encoder in the BERT MLM model are visualized in two-dimensional space with T-distributed Stochastic Neighbor Embedding (t-SNE). Figure 4 shows embedding for different symbols, we can see that the embedding obtained by the 2-encoder tended to merge symbols have similar meaning together compared to BERT MLM (for example, the embedding of ’1’, ’2’, ’3’, ’4’, ’5’ are overlapped together for 2-encoder model, whereas BERT MLM divided these symbols into parts; embedding of ’s’ and ’o’ are closer to each other for 2-encoder compared to BERT MLM; for symbols used to indicate stereo structure, ’$\backslash$’ and ’/’ are more closer for 2-encoder model compared to BERT MLM, ’@’ and ’@@’ are overlapped with ’H’ for 2-encoder while BERT MLM separated them into parts). Then molecular embedding which is the output of the first encoder corresponding to ’cls’ for the 2-encoder model and the results of average pooling symbol embedding for BERT MLM are visualized as shown in Fig. 5. We can see the 2-dimensional distribution of molecular embedding obtained by BERT MLM is clearly dominated by the number of benzene rings and the molecular weight. (From top to bottom the number of benzene rings in the molecule increases, and from left to right the molecular weight increases.) And the visualization of molecular embedding for the 2-encoder model shows that the 2-encoder model enables molecules without benzene rings to cluster together, however, it does not show significant distribution trend in the 2D visualization according to the number of benzene rings and molecular weight in general. The molecular embedding obtained from pretrained model dominated by certain features of molecules (the number of benzene rings and molecular weight in the BERT MLM case) may have a positive or negative impact depending on the task of transfer learning.

### Results of molecular property prediction

For both the BERT MLM and 2-encoder model, fine-tuning only the parameters of the last encoder layer and using two fully connected layers as the classifier layer provided the best predictions of molecular properties.

**Table 3:** Prediction results of 22 ADMET data sets

	Task type	Sample size	Metric	2-encoder	BERT MLM	non-pretrain
ames	Classification	7278	AUROC	**0.829**	0.818	0.754
bbb_martins	Classification	1975	AUROC	**0.881**	0.875	0.846
bioavailability_ma	Classification	640	AUROC	0.605	**0.749**	0.669
caco2_wang	Regression	910	MAE	**0.348**	0.372	0.423
clearance_hepatocyte_az	Regression	1213	Spearman	**0.435**	0.396	0.363
clearance_microsome_az	Regression	1102	Spearman	**0.633**	0.518	0.375
cyp2c9_substrate_carbonmangels	Classification	669	AUPRC	0.336	0.377	**0.38**
cyp2c9_veith	Classification	12092	AUPRC	**0.758**	0.739	0.68
cyp2d6_substrate_carbonmangels	Classification	667	AUPRC	**0.722**	0.608	0.581
cyp2d6_veith	Classification	13130	AUPRC	**0.656**	0.631	0.584
cyp3a4_substrate_carbonmangels	Classification	670	AUROC	**0.655**	0.645	0.575
cyp3a4_veith	Classification	12328	AUPRC	0.843	**0.847**	0.78
dili	Classification	475	AUROC	**0.872**	0.838	0.852
half_life_obach	Regression	667	Spearman	0.088	**0.405**	0.149
herg	Classification	655	AUROC	0.793	0.775	**0.836**
hia_hou	Classification	578	AUROC	0.98	**0.984**	0.98
ld50_zhu	Regression	7385	MAE	**0.583**	0.683	0.635
lipophilicity_astrazeneca	Regression	4200	MAE	**0.586**	0.613	0.802
pgp_broccatelli	Classification	1218	AUROC	**0.929**	0.885	0.903
ppbr_az	Regression	2790	MAE	**8.578**	8.697	9.081
solubility_aqsoldb	Regression	9982	MAE	0.899	**0.838**	0.907
vdss_lombardo	Regression	1130	Spearman	0.505	**0.545**	0.478

Numbers in bold indicate the best results among the three models

The prediction results for the 22 data sets are shown in Table 3, and the results corresponding to more metrics and the current ranking based on the leaderboard provided by TDC can be found in the additional file. Among three models of 2-encoder, BERT MLM and non-pretrain, 2-encoder achieves the best results on 14 data sets, BERT MLM achieves the best results on 6 data sets, non-pretrain achieves the best results on 2 data sets. And for most of the tasks, the pretrained model achieved better results than non-pretrain. Moreover, there are three notable observations in the comparison of the three models. (1) For data sets of ’clearance_microsome_az’, ‘clearance_hepatocyte_az’, ‘cyp2d6_substrate_carbonmangels’, ‘ld50_zhu’ and ‘cyp3a4_substrate_carbonmangels’, the results of 2-encoder are ranked in top 3 on the leaderboards. And for the first three data sets in these four data sets, the 2-encoder can achieve apparently better results than BERT MLM and non-pretrain. For ‘ld50_zhu’, BERT MLM is worse than non-pretrain. (2) For the data set ‘bioavailability_ma’, BERT MLM achieved much better results (was ranked first according to the leaderboards) and 2-encoder was ranked last. The results on data set ‘half_life_obach’ also show a similar tendency. (3) For the data sets ‘cyp2c9_substrate_carbonmangels’ and ‘herg’, non-pretrain achieved the best results among three models, and 2-encoder was ranked last for the data set ‘cyp2c9_substrate_carbonmangels’.

The observations of results show that the benefits of two kinds of pretraining strategies are not consistent with different tasks. As mentioned in [17], the pretraining model focusing on node-level and graph-level will have different effects according to tasks of transfer learning, and even sometimes pretraining can have negative effects since the tasks for pretraining and transfer learning are unrelated. Our experimental results show a similar phenomenon. The visualization of symbol embedding (Fig. 4) shows that symbol embedding with similar meaning in SMILES are closer even overlapped together for the 2-encoder model, which can be considered as the pretraining of the 2-encoder model introduced more bias induction to symbols. And the visualization of molecule embedding (Fig. 5) shows that the pretraining of BERT MLM introduced more bias induction of properties such as molecular weight to molecular embedding compared to the 2-encoder. However, these bias induction may not necessarily be associated with the task of transfer learning. For example, the visualization of symbol embedding for the 2-encoder model (Fig. 4) shows that the symbol ’H’ is mixed with ’@’ and ’@@’ together since ’H’ appears simultaneous with ’@’ frequently, however, in the actual molecular structure, hydrogen atoms are not highly associated with chiral atoms. We assumed it is possible to introduce an incorrect bias induction to the pretrained model since the limitation of BERT MLM(the surrounding symbols of masked symbols can’t determine the masked symbols).

In addition, prediction results recorded on the TDC leaderboards show that models based on random forest with inputs of molecular fingerprints and 200 molecular properties computed by RDKit can achieve better results than 2-encoder, BERT MLM and non-pretrain on most data sets. This may be due to the stronger correlation between inputs and the 22 ADMET tasks, which open our eyes to the possibility of improving the bias induction introduced by pretraining to be more comprehensive by pretraining model with fingerprints and 200 molecular properties.

**Table 4:** F1 results of 98 ODS predictions

	Macro F1	Micro F1
non-pretrain	0.242	0.340
BERT MLM	0.260	0.357
2-encoder	0.294	0.390

The prediction results for 98 ODs are given in Table 4. As a complex and comprehensive task of predicting 98 kinds of OD simultaneously, the pretrained model achieved better results than non-pretrain, and the pretrained by the 2-encoder model can achieve better results than BERT MLM.

In summary, even though the benefits of pretraining vary according to different tasks, the 2-encoder pretraining model achieves better results than BERT MLM and non-pretrain in more than half of tasks, which can show that the pretraining model with a 2-encoder structure is more robust than the widely used BERT MLM.

## Conclusion

We proposed a pretraining model for predicting molecular properties using SMILES sequences which can fully represent the molecular structure. On the basis of the pretraining model BERT used in natural language processing, we proposed an unsupervised pretraining model for learning the molecular structural information included in the SMILES sequence before predicting the specific molecular properties. In an experiment, we pretrained the proposed model and then predicted 22 molecular properties and 98 ODs. The visualization of embedding obtained through the pretraining model with 2-encoder and BERT MLM shows the different bias induction on the symbol level and SMILES level. Even these inductive bias effect transfer learning differently according to tasks, the proposed 2-encoder model outperformed the non-pretrained model and the BERT MLM on more than half of tasks.

### Supplementary Information

**Additional file 1.** Supplementary Table.

## Data Availability

The experimental code is provided at https://github.com/zhenghah/230502 .

## Abbreviations

SMILES: Simplified molecular-input line-entry system
OD: Odor descriptor
NSP: Next sentence prediction
MLM: MLM masked language model
